# Genome-wide survey of allele-specific splicing in humans

**DOI:** 10.1186/1471-2164-9-265

**Published:** 2008-06-02

**Authors:** Victoria Nembaware, Bukiwe Lupindo, Katherine Schouest, Charles Spillane, Konrad Scheffler, Cathal Seoighe

**Affiliations:** 1 Institute of Infectious Disease and Molecular Medicine, University of Cape Town, Private Bag, Rondebosch, 7700, Cape Town, South Africa; 2 Genetics & Biotechnology Lab, Department of Biochemistry & BioSciences Institute, University College Cork, Cork, Ireland

## Abstract

**Background:**

Accurate mRNA splicing depends on multiple regulatory signals encoded in the transcribed RNA sequence. Many examples of mutations within human splice regulatory regions that alter splicing qualitatively or quantitatively have been reported and allelic differences in mRNA splicing are likely to be a common and important source of phenotypic diversity at the molecular level, in addition to their contribution to genetic disease susceptibility. However, because the effect of a mutation on the efficiency of mRNA splicing is often difficult to predict, many mutations that cause disease through an effect on splicing are likely to remain undiscovered.

**Results:**

We have combined a genome-wide scan for sequence polymorphisms likely to affect mRNA splicing with analysis of publicly available Expressed Sequence Tag (EST) and exon array data. The genome-wide scan uses published tools and identified 30,977 SNPs located within donor and acceptor splice sites, branch points and exonic splicing enhancer elements. For 1,185 candidate splicing polymorphisms the difference in splicing between alternative alleles was corroborated by publicly available exon array data from 166 lymphoblastoid cell lines. We developed a novel probabilistic method to infer allele-specific splicing from EST data. The method uses SNPs and alternative mRNA isoforms mapped to EST sequences and models both regulated alternative splicing as well as allele-specific splicing. We have also estimated heritability of splicing and report that a greater proportion of genes show evidence of splicing heritability than show heritability of overall gene expression level. Our results provide an extensive resource that can be used to assess the possible effect on splicing of human polymorphisms in putative splice-regulatory sites.

**Conclusion:**

We report a set of genes showing evidence of allele-specific splicing from an integrated analysis of genomic polymorphisms, EST data and exon array data, including several examples for which there is experimental evidence of polymorphisms affecting splicing in the literature. We also present a set of novel allele-specific splicing candidates and discuss the strengths and weaknesses of alternative technologies for inferring the effect of sequence variants on mRNA splicing.

## Background

One of the key tasks of the post-genome era is to determine the functional implications of genomic variants. The development of high throughput genotyping technologies and the use of these technologies in large-scale studies has enabled the identification of increasing numbers of human loci that are associated with common genetic disorders (e.g.[1]). However, the mechanisms through which genetic variants at many disease-associated loci affect disease susceptibility remain to be determined. Mutations or polymorphisms that affect mRNA splicing can have a profound effect on the function of the spliced product, but these effects are often difficult to predict from the primary genomic sequence. The medical and biological significance of such variants is evident from the large and rapidly increasing volume of literature reporting examples of aberrant mRNA splicing associated with human cancers and genetic diseases [2,3]. Indeed, point mutations leading to aberrant splicing are thought to be among the most important contributors to human genetic diseases [4].

Sequence variants found on the pre-mRNA can affect a number of different, and in some cases imperfectly characterized, *cis*-acting sequences that control splicing. Polymorphisms that occur at the highly conserved donor and acceptor di-nucleotides are an obvious case in which we expect an effect on splicing [5] and these genomic variants, when they occur close to verified exon boundaries, tend be annotated in databases of sequence polymorphisms, such as dbSNP [6]. A much larger proportion of variants are likely to occur at sites where the effect on splicing is less obvious, for example at less conserved sites close to intron/exon boundaries, close to the intronic branch-point [7], or within intronic or exonic splicing enhancer or suppressor sequences [8]. In some cases, such sequence variants disrupt normal gene splicing, causing aberrant splicing of either a proportion, or all of the transcripts produced. However, if the gene is alternatively spliced to begin with, then sequence variants that affect sites that are involved in controlling isoform abundance may be affected, causing allelic differences in the regulation of alternative splicing, with potentially important biological consequences [9].

The contribution of heritable variation to the observed diversity of mRNA splice isoforms is well established [10-12]. Using the ASAP database of alternatively spliced mRNA isoforms [13] and transcribed SNPs, we previously estimated that approximately 20% of alternatively spliced genes show evidence of allele-specific splicing (either complete allele-specific splicing, in which one allele gives rise to one isoform and another results in the alternative form, or partial allele-specific splicing in which different alleles result in distinct relative isoform abundance [10]). Earlier large-scale studies of alternative and allele-specific splicing relied primarily on Expressed Sequence Tag (EST) sequences. More recently, both exon-junction and exon tiling arrays have been used for genome-wide studies of alternative splicing [14,15]. The Affymetrix GeneChip Human Exon 1.0 ST Array has probe-sets targeting approximately 1.4 million known and predicted exons. Alternatively spliced mRNA isoforms detected using the Affymetrix exon array in cell lines genotyped as part of the HapMap project [16], has given rise to opportunities for high-throughput discovery of alleles that affect mRNA splicing [11,12]. Though exon arrays are arguably a superior technology, with better exon coverage than ESTs [12], they are also affected by a range of caveats that need to be considered [17]. Integration of results from ESTs and microarrays is likely to increase power to detect allele-specific splicing as both arrays and ESTs have different limitations and advantages for the analysis of alternatively spliced isoforms.

Though for the present it remains a distant goal, a complete description of the effect of human sequence variants on mRNA splicing would be a powerful resource for understanding human genetic diseases and phenotypes. One option for evaluating the potential effect of *cis*-acting mutations on splicing is to use *ab initio *prediction algorithms that make use of the availability of the complete human genome sequence [18,19]. In several previous studies, computational tools have been effective in helping to shed light on the impact of a mutation on splicing [9,20,21] and databases of mutations that may affect splicing have been made available [22,23]. However, because of the difficulty of predicting all splice regulatory elements from genomic sequence and the even greater difficulty of determining accurately the effect of mutations in these regions on splicing, genomic analysis of SNPs likely to affect splicing needs to be complemented by expression data that provides information about the splice isoforms that are associated with the alternative alleles of a candidate SNP.

We have performed a genome-wide scan for Single Nucleotide Polymorphisms (SNPs) likely to influence splicing efficiency in *cis *using publicly available tools (ESEfinder, [19], MaxENTScan [18], and Branch Site Analyzer [24]). We have tested predictions based on genomic sequences using publicly available EST and exon array data. We present a novel probabilistic method to infer allelic differences in mRNA splicing from EST data, and use recently published Affymetrix exon array hybridisation data derived from 166 lymphoblastoid cell lines [25] for which genome-wide genotype data are available through the HapMap project [16] to test for association between mRNA isoforms and the genotype of putative *cis-*acting splicing polymorphisms. We have also investigated the heritability of splicing and compared it to heritability of transcript level expression using the exon array data.

## Results

### A genome-wide scan for polymorphisms in splice-regulatory regions

We used published computational tools and identified 30,977 polymorphisms that occur within predicted or known splicing regulatory sequences (srSNPs), including donor sites, acceptor sites, branch points (BP) and exonic splice enhancer (ESE) elements [see Additional file 1]. The number of SNPs occurring in putative ESEs is much higher than the number in the other *cis *elements (Table 1). This is likely to be due, at least in part, to the high false positive rate of ESE identification compared to the other splice regulatory elements that are identified using positional information, rather than by matching to sequence patterns alone. For each type of splice-regulatory element, publicly available tools were used to score the sequences associated with alternative SNP alleles (see Methods) and score differences for the identified polymorphisms are reported as supplementary data. We used gene structure information from Ensembl [26] as well as from ASAPII [27], to identify srSNPs. This greatly increased our coverage, for example, of the 9,201 polymorphisms identified in donor and acceptor regions (including 17 in dual-specificity sites [28]), 3,868 occurred within exon-intron or intron-exon boundaries common to both databases while 2,759 were unique to Ensembl and 2,574 were unique to ASAPII.

**Table 1:** Summary of srSNPs with supporting evidence from EST and Exon array data.

***Cis*element**		**srSNPs**	**EST evidence (α = 0.05)**	**Exon array (FDR = 0.1)**	**Exon array (Holm corrected α = 0.05)**
Donor		1970	47	84	20
Acceptor		7248	156	217	22
Branch		2689	41	75	13
ESEs	SC35	5910	44	257	26
	SF2	8992	82	387	44
	SRp40	7776	94	334	32
	SRp55	5231	64	211	19

### A maximum likelihood method to identify allele-specific splicing using EST data

**Figure 1:**
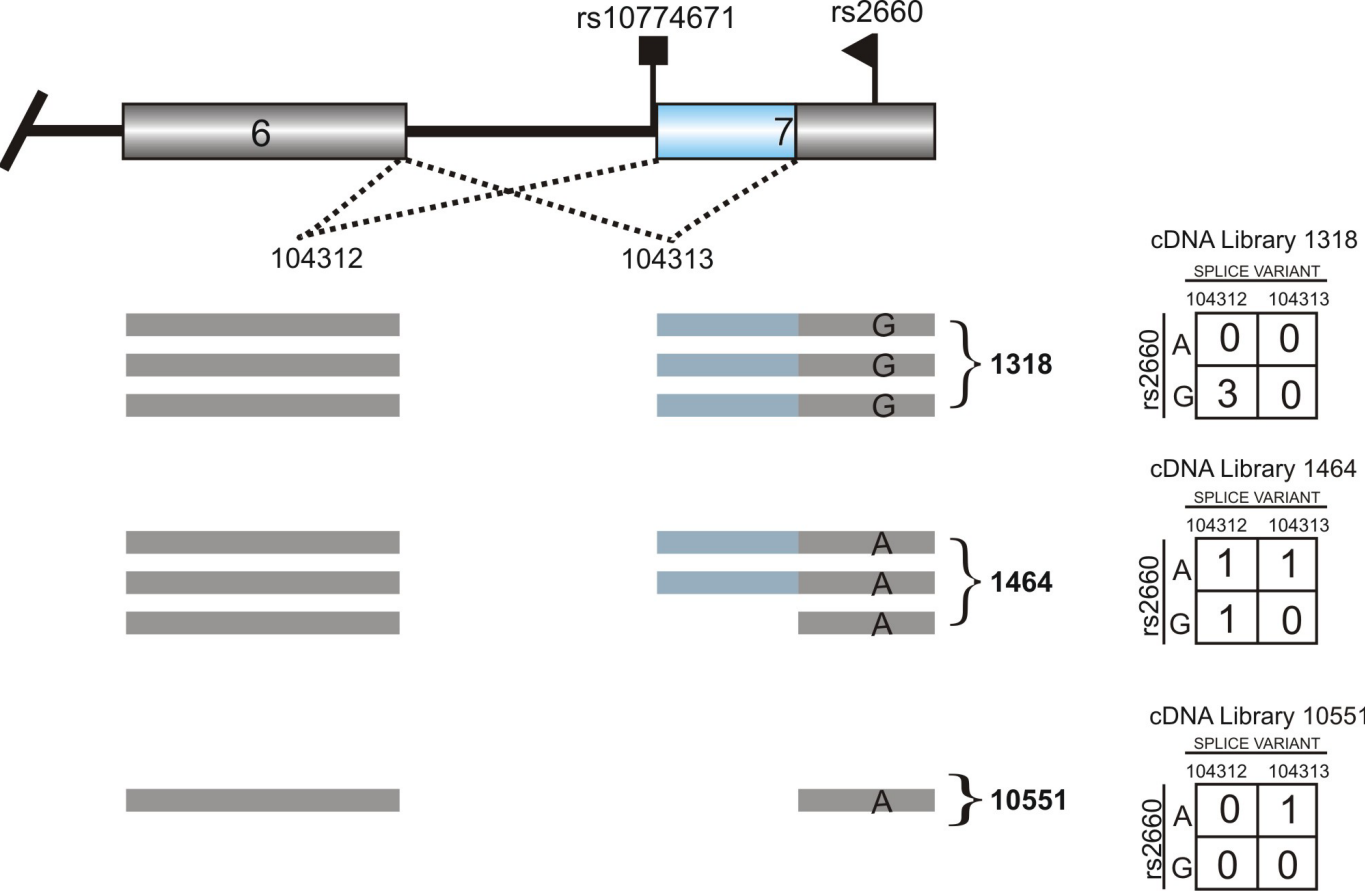
**Data used to detect mSNPs**. Part of the genomic sequence of the *OAS1 *gene showing alternative acceptor site use at exon 7. The putative causative SNP (rs10774671), which occurs at the G site of the canonical acceptor dinucleotide, and an mSNP (rs2660), which was used to infer allele-specific splicing from EST data, are shown. Splice isoforms and mSNP alleles observed in three of a total of 27 cDNA libraries with ESTs that mapped to this region are also depicted. For each library the data are summarized in a two-by-two contingency table, with each ESTs cross-classified according to mRNA isoform and SNP allele.

We previously used linkage disequilibrium between SNPs mapped to EST sequences and alternative splice isoforms to identify allele-specific mRNA isoforms [10]. However, because alternative splicing can be regulated in a tissue-specific manner and because multiple ESTs from the same gene can occur in a single cDNA library, we restricted our previous analysis to just one EST per cDNA library per alternative isoform pair. To make better use of the available data we have now developed a probabilistic model that can be applied to detect allele-specific splicing from SNPs mapped to EST sequences (see Methods and an illustration of the data for an example gene in Figure 1). The possibility that the isoform is regulated in a tissue-specific manner is modeled explicitly. For a given pair of mutually exclusive mRNA isoforms, the proportions of each isoform that occur across different cDNA libraries are modeled using a beta distribution. An allelic effect on splicing is inferred when a model that allows separate beta distributions for two alternative alleles of a SNP (which maps to both isoforms) provides a better fit to the data than a model with a single distribution for both alleles. We found 1,753 marker SNPs (i.e. SNPs in linkage disequilibrium with a splicing event, which we refer to as mSNPs), corresponding to 1,318 genes and 2,283 alternative splice junction pairs, for which the allele-specific mRNA splicing model provided a better fit to the data than the null model at the 5% significance level, using the likelihood ratio test [see Additional file 2].

**Figure 2:**
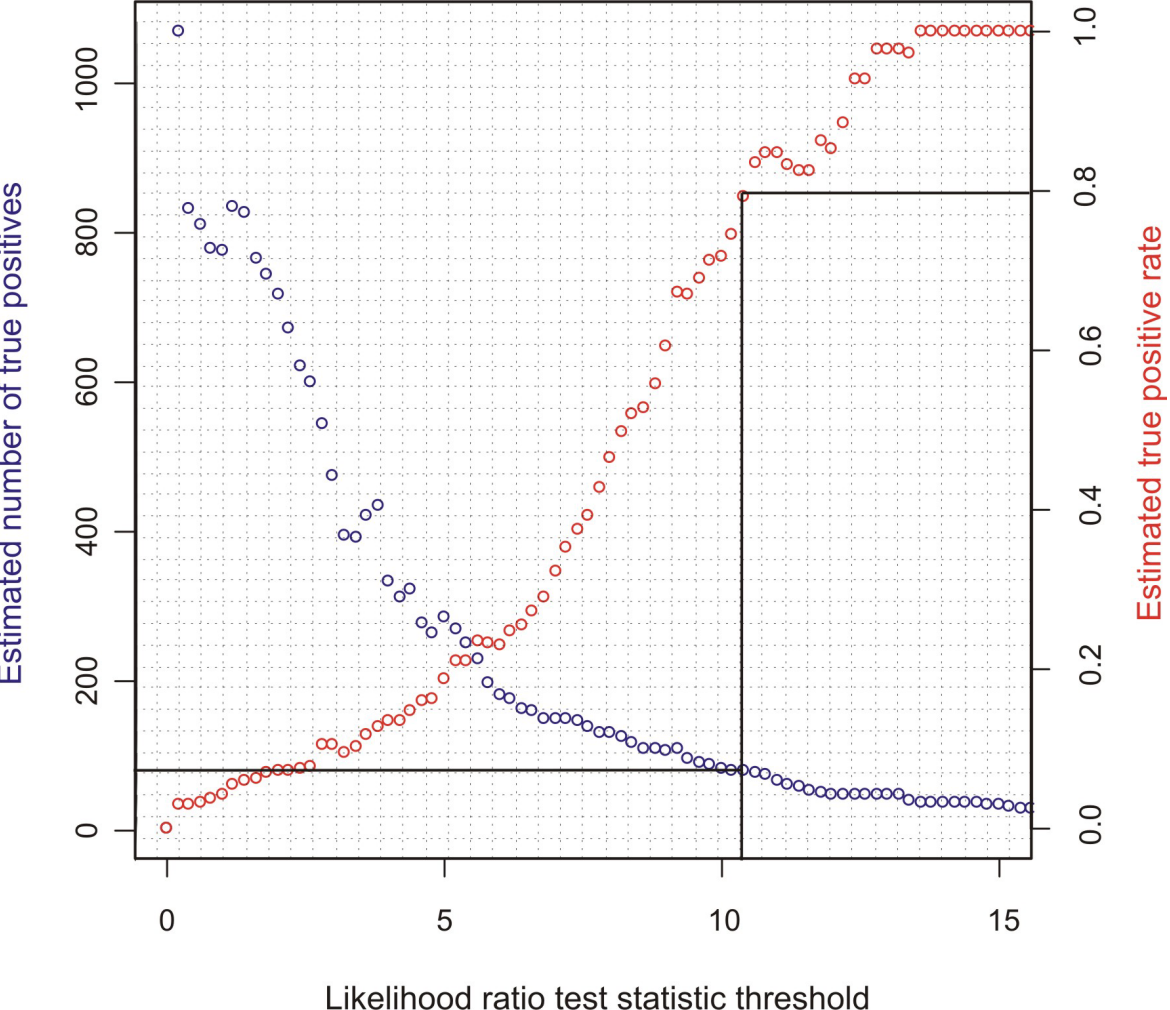
**Analysis of simulated EST data**. Results of analysis of randomized EST data. The number of true positives and the true positive rate (equal to one minus the false discovery rate) as a function of the likelihood ratio test statistic were estimated from 1000 randomizations of the matrices of counts of ESTs mapping to alternative SNP alleles and alternative splice isoforms. The solid line shows the number of true positives obtained when the true positive rate is 0.8 (i.e. at a false discovery rate of 0.2).

The distribution of the likelihood ratio test statistic is asymptotically chi-squared for large sample sizes under the null hypothesis. To test the validity of the test on the observed data, for which the number of data points per test was highly variable, we simulated data identical to the observed data in terms of the numbers of ESTs mapping to alternative alleles and splice isoforms but conforming to the null hypothesis of no association between mRNA isoform and allele. The cumulative distribution of the likelihood ratio statistic on this simulated data was consistently lower than the chi-squared distribution with one degree of freedom (data not shown), which suggests that the likelihood ratio test provides a conservative basis on which to reject the null hypothesis. The distribution of p-values from the simulated data was also not uniform because of the sparseness of the data available for many of the mSNP and splice junctions that were tested. This complicates the application of standard false discovery rate methods to account for multiple testing. Instead we compared the observed and simulated distributions of the likelihood ratio test statistic, which allowed us to estimate the proportion of false discoveries at all levels of the test statistic (Figure 2). There were 91 cases of association between mSNPs and splice isoforms at the true positive rate cut-off of 0.8, shown on the graph (corresponding to approximately 73 true positives and 18 false positives; Figure 2). These came from 54 distinct alternate splice junction pairs and 51 different genes.

### Support for srSNPs and mSNPs from publicly-available exon array data

**Figure 3:**
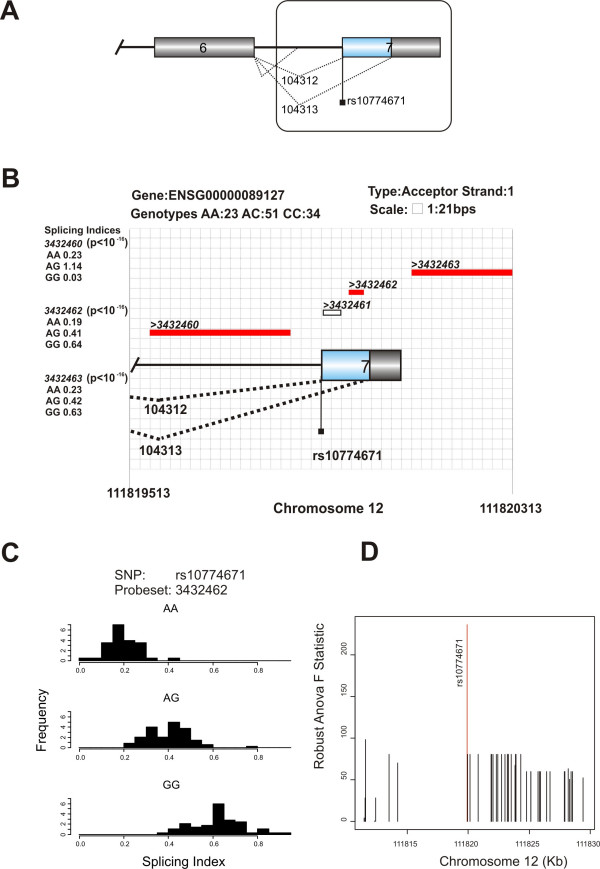
**Allele-specific splicing evidence in the OAS1 based on exon array analysis**. Support for allele-specific acceptor site use in the *OAS1 *gene. (A) Genomic sequence of OAS1 showing the alternatively spliced exons. The boxed section is magnified and drawn to scale in the next panel. (B) Relationship between the genotypes of the SNP and splicing indices of nearby probesets, illustrating that there is likely to be a complex pattern of allele-specific splicing in this gene. Probesets in red are significantly associated with the SNP genotype. The p-values for the association of these probesets to SNP genotypes are also included. Unfilled rectangles represent probesets that were not tested for an association with the genotype because they were not detected above background in a sufficient number of the cell lines, or were too distant from the SNP. (C) Histograms showing the splicing index distribution as a function of the genotype of a SNP, rs10774671, at the G nucleotide of the canonical splice acceptor site. (D) Association plot illustrating that rs10774671 is more strongly associated with a probeset between the SNP and an alternative acceptor site than any other SNP in the region for which genotype data were available.

**Figure 4:**
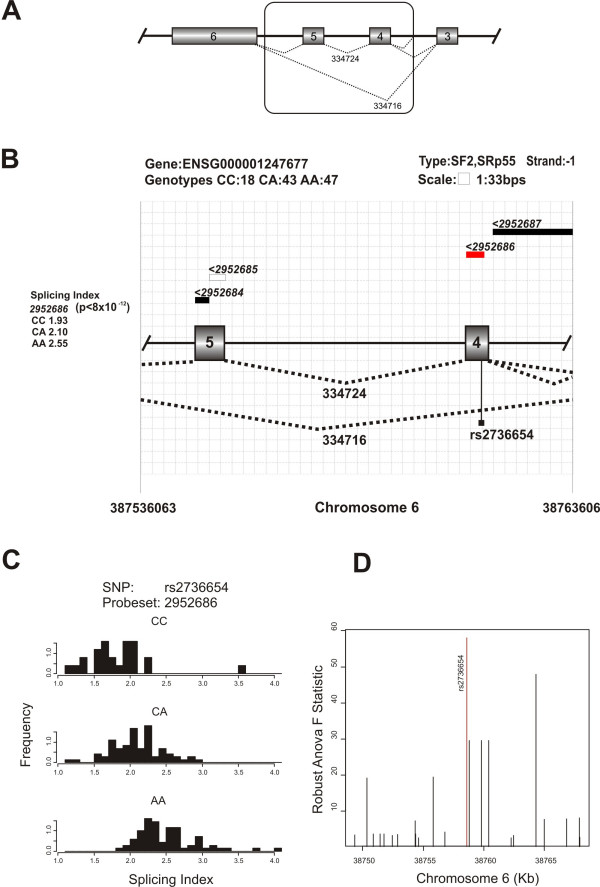
**Allele-specific splicing evidence in the *GLO1 *based on exon array analysis**. Support for allele-specific exon-skipping in the *GLO1 *gene. (A) Genomic sequence of the *GLO1 *gene showing the alternatively spliced exons. The boxed section is magnified and drawn to scale in the next panel. (B) Illustration of the relationship between the genotypes of this SNP and splicing indices of nearby probesets, using the same conventions as in Figure 3. (C) Histograms showing the splicing index distribution as a function of the genotype of a SNP, rs2736654, predicted to affect an exonic splice enhancer site. (D) Association plot illustrating that rs2736654 is marginally more strongly associated with a probeset spanning exon 4 than any of the other SNPs in the region for which genotype data were available.

Exon array data generated by Huang *et al*. [25] from 166 lymphoblastoid cell lines using the Affymetrix Exon 1.0 ST array were downloaded from the GEO database [29], and processed as described in Methods. The splicing index (SI; [30]) was calculated for each probeset (including high-confidence core probesets as well as probesets corresponding to predicted exons), by dividing the probeset-level expression estimate by the meta-probeset (or transcript) level expression estimate. The transcript-level expression estimates were inferred using core probesets only, to avoid inaccuracy caused by including spurious probesets in the transcript-level expression estimate [12]. Genome-wide genotype data for almost four million SNPs were available for the same cell lines through the HapMap project [23]. For each putative srSNP and mSNP for which genotype data were available we tested for an effect of genotype on SI for each probeset in the region of the mSNP or srSNP, treating the HapMap population from which the sample was derived (Yoruban or Caucasian) as a covariate, and using a robust linear model and robust analysis of variance (ANOVA), implemented in the Insightful Robust Library of the R package [31,32]. In the case of srSNPs, because it is often difficult to predict the impact of the SNP on splicing, all probesets within 1 kb of the SNP were tested. For the mSNPs we tested only probesets that fell within the genomic boundaries defined by the alternative exon junctions of the putatively allele-specific splice isoforms (i.e. probesets with genomic coordinates between the minimum and maximum of the genomic coordinates of the exon boundaries implicated in the alternative splicing event). Similarly, to determine whether an srSNP was supported by EST data, we tested whether the srSNP fell within the genomic region defined by the alternative exon junctions (including 3 bp of the corresponding exons in the case of putative exonic splice donor and acceptor mutations). Examples of srSNPs for which there was strong evidence of an allelic effect on splicing from the exon array data (Holm-corrected p-value < 0.05) are shown in Figures 3 and 4. Similar diagrams are available for a total of 1,185 putative srSNPs for which there was a probeset SI significantly associated with genotype [see Additional file 3].

Among the classes of splicing regulatory regions analysed, SNPs that occurred in donor sites were slightly more likely to be confirmed by EST and/or exon array data (Table 1). In addition to the srSNPs for which there is supporting evidence from EST and/or exon array data a further 66 mSNPs were supported by exon array data, but no candidate srSNP was identified that could explain the allelic difference in splicing. Some of these may be false positive mSNPs but for the remainder, the causative SNP may be in an intronic splicing element (intronic splicing elements were not included in the genome-wide scan for srSNPs) or in, as yet, uncharacterized splicing regulatory elements. The possibility also exists that some of the identified putative allele-specific isoforms are caused by mutations located within *trans *regulators of splicing and that association with nearby polymorphisms is a result of population stratification rather than a direct *cis*-acting effect.

### Cross-validation of EST and exon array results

Of the 54 distinct alternate exon junction pairs with evidence of allele specific splicing from the EST data using a false detection cut-off of 0.2 (see above), 15 could be tested for allele-specific splicing using the exon array data (in order to be tested the mSNP had to be among the SNPs genotyped in the HapMap populations, a probe or probes had to occur between the genomic coordinates spanned by the alternative exon junction pair and the probe had to be detectable above background in at least some of the cell lines). Of these 15, 9 (60%) had at least one probe between the genomic coordinates of the junction pair for which the SI was significantly associated with the genotype of the mSNP (p < 0.05, with Bonferroni correction in the case where multiple probes were tested for association with a single mSNP). By comparison, for a random set of 10,000 alternatively spliced exon junction pairs from ASAPII and nearby exonic SNPs that showed no association with the mRNA isoform there were 252 (22%) associations from 1,167 exon junctions that could be tested. The proportion of allele-specific splicing candidates from the EST data that could be confirmed using the exon array data was significantly higher than for alternatively spliced exon junctions with no evidence of allele-specificity from ESTs (p = 0.002 using Fisher's Exact Test). This overlap of allele-specific splicing candidates identified by very different technologies, provides cross-validation for the candidates identified using the two approaches.

### Splicing index association plots

A significant association between the SI of a probeset and an srSNP is insufficient to infer a causal relationship between the srSNP and variation in SI. It is possible that the putative srSNP is not causally related to the observed difference in splicing and, instead, that it is in linkage disequilibrium with a nearby SNP that was not predicted to affect splicing (because of the imperfect understanding of splicing regulation). We can begin to investigate this possibility by testing for an association between the SI and genotype for all of the other nearby SNPs for which genotype data are available for the lymphoblastoid cell lines. For each srSNP we tested for an association between SI and genotype for all genotyped SNPs within 10 kb of the srSNP. On average there were 25 such SNPs per srSNP. For the majority (61.8%) of the srSNPs supported by the exon array data (fdr < 0.1), the predicted srSNP showed the most significant or joint most significant association between SI and genotype for at least one of the probesets tested. For the remainder, an alternative SNP, not necessarily predicted to affect splicing, showed a more strongly significant association. The mechanisms through which these alternative SNPs may affect splicing require further investigation. Examples of the association plots are shown in Figures 3 and 4. Similar association plots for all of the srSNPs supported by the exon array data are available from .

### Analysis of allele-specific mRNA splicing candidates

For several of the examples of allele-specific splicing that we identified we were able to find published research articles confirming the same event (Table 2). The mSNP rs2660 (p = 0.0006; Figure 1), which we detected in the 2',5'-oligoadenylate synthetase 1 (*OAS1*) gene, for example, has been shown experimentally to be in strong linkage disequilibrium with the srSNP, rs10774671, which occurs at the G of a canonical acceptor site [33]. Disruption of the canonical acceptor site in intron 6 of the *OAS1 *gene promotes the use of two cryptic acceptor sites. Using the EST data we detected one of the cryptic acceptor sites, located 98 bps from the wild type acceptor site (Figure 1). This event was also detectable using the exon array data (Figure 3).

**Table 2:** A subset of the previously reported allele-specific splice isoforms detected in this study

**Gene**	**Exon**	**mSNP (p-value)**	**mSNP (p-value from exon array data)**	**srSNP (exon-array p-value)**	***Cis*-element**	**References**
*CD45*	Exon 4	rs12129883 (0.020551)	---	---	ESS	[54]
*COL5A1*	Exon 65	rs13946 (0.046)	---	---	Acceptor site	[55]
*ETV4*	Exon 3	rs3765174 (0.014)	---	---	NAGNAG acceptor	[39]
*GABRR1*	Exon 2	rs12200969 (0.034)	---	rs4590242-NA-	NAGNAG acceptor	[39]
*ITPA*	Exon 2 and Exon 3	rs13830 (0.030)	0.0042	---	Exonic splicing silencer element in exon 2	[56]
*MUC1*	Exon 2	rs4072037 (0.0011)	---	---	Acceptor site	[57]
*OAS1*	Exon 7	rs2660 (0.00063)	2.78 × 10^-15^	rs10774671 (< 10^-16^)	Acceptor	[33]
*PMM2*	Exon 5	rs2072688 (0.0027)	---	---	ESE	[44]
*RBM23*	Exon 6	rs1951119 (1.0 × 10^-6^)	0.0140	rs2295682 (0.0027)	SRp40*	[11]
*UROD*	Exon 4	rs1804886 (0.0027)	---	---	---	[58]

* Hull *et al*. [11] did not report that the SNP, rs2295982, disrupts an ESE

There are also many cases of previously unpublished splicing polymorphisms among our results, some of which are likely to be functionally and medically important. For example, the exon array data provide strong evidence (robust ANOVA F statistic: 40.5; p = 9 × 10^-11^) for an association between a probeset in exon 4 of the *GLO1 *gene, encoding an enzyme (glyoxalase I) that has been reported to show lower activity in the brains of individuals affected by autism compared to control individuals [34] and the genotype of a SNP in the same exon (C419A or rs2736654; Figure 4). Reduction in enzyme activity has been attributed to the direct effect of this non-synonymous SNP on the amino acid sequence of the protein. The ancestral A allele has been reported to be significantly associated with autism [34] and certain types of panic disorders [35]. A larger scale study, however, has questioned the association with autism, but has found that the A allele may have a protective effect in the siblings of individuals with autism [36]. This non-synonymous SNP occurs in a predicted exon splice enhancer site (the genomic scan for srSNPs predicts that this site acts as an ESE for both SF2 and SRp55 and the A and C alleles have scores 0.44 and 2.96, respectively, for SF2 and 1.39 and 3.53, respectively for SRp55). EST evidence from ASAPII suggests that two exons are skipped [27]. Skipping of these exons is likely to have a much greater impact on the protein function than the replacement of alanine by glutamine at a single site within one of the exons. While the role of *GLO1 *in neurological disorders remains controversial [37], Sacco *et al*. [36], highlight the need for further investigation of the functional impact of the C419A. Our results suggest that the polymorphism is very likely to impact on splicing. This could have a significant impact on glyoxalase I activity and be the mechanism underlying the disease association.

### Heritability of splicing

We assessed the heritability of splicing index by estimating the slope of the correlation of child SI with the mean of parent SI values for 46 two-parent and child trios, for which exon array data were available for the complete trio. Using only core probesets and meta-probesets, the mean of the slope as well as the mean of Pearson's correlation coefficient were positive (0.093 and 0.084, respectively) and significantly different from zero (p < 10^-16 ^in each case), but significantly less than the mean values obtained when we performed the same regression using estimated transcript-level (i.e. meta-probeset) expression values, for which the corresponding values were 0.163 and 0.146. We estimated the proportion of SI values that do not conform to the null hypothesis (which assumes no correlation between mean parent SI and child SI) using the method described by Storey and Tibshirani [38]. This proportion was 15% for splicing index, rising to 35% for transcript-level expression estimates. This suggests that probeset splicing index is, on average, somewhat less heritable than whole transcript expression. However, because probeset expression levels are estimated from much smaller numbers of probes than transcript level expression, probeset expression level estimates are likely to be noisier and this may contribute to their apparently lower heritability. It is also important to note that there were approximately 13 probesets per core meta-probeset. At the 1% significance level, 4.8% of probesets were correlated between child and the mean of the parent values. This figure was 7.7% for meta-probeset expression level estimates, but 12.3% of genes had at least one probeset with a significantly correlated SI value (applying Bonferroni correction for multiple probesets per meta-probeset). Therefore, heritability of the relative expression of parts of transcripts appears to be common, even more so than heritability of overall gene expression level.

## Discussion

Large-scale discovery of genomic variants that affect splicing has the capacity to accelerate the association of diseases to causative genomic variants. However, because it is difficult, and in many cases currently not possible, to determine the effect of a genomic variant on splicing or on the regulation of alternative splice isoforms from genomic sequence data alone, this remains a challenging task and requires the integration of information from different data types. At present, no single source of data can provide information about all forms of splice variants and each source of data has advantages as well as disadvantages. The publicly available exon array data that we have used here represents an extremely extensive dataset on isoform abundance in human lymphoblastoid cell lines that can be correlated with the genotype of the cell line. However, this data provides no information on transcripts that are not expressed in lymphoblastoid cell lines, or on splicing mutations that affect relative isoform abundance in only a subset of expression contexts. Furthermore, depending on the exact location of probesets in a given gene, many of the transcript isoforms that occur, particularly those that affect donor or acceptor site but do not cause exon skipping or inclusion, are undetectable using exon arrays. When alternative isoforms are distinguishable using the exon arrays, they still provide little information on the nature of the isoforms, and this may need to be inferred either by integrating information from other sources or experimentally.

EST sequences provide information on the structure of alternative isoforms and include data from different gene expression contexts, but this information is highly biased towards ends of genes and is sparse, for all but the most highly expressed genes. The simulations provide ample evidence for frequent allele-specific splicing but also illustrate that there is not enough data to confirm most cases, especially when the effect of a very large number of statistical tests is considered. There were several published examples in the current study of genes known to be spliced in an allele-specific manner, but for which the allele-specific splicing model fits the data no better than the null model. However, although most cases of allele-specific splicing will not be detectable using EST sequences alone, ESTs can often be used to elucidate the nature of the allele-specific splicing events detected because ESTs provide information on the actual transcripts that occur. We compared the length differences between alternatively spliced isoform pairs with and without evidence of allele specific splicing from the EST analysis. Across all classes of alternative splicing considered the length differences between isoform pairs were smaller for pairs with evidence of allele-specific splicing. There was also evidence of a trend towards a greater likelihood of frame preservation among the isoforms with evidence of allele specificity [see Additional file 4].

*GLO1 *provides an example of a gene with a mutation that is likely to affect splicing, but although there was good coverage of this gene in the EST databases, the allele-specific splicing event was not detectable from the EST data. Because the putative causal SNP is on the skipped exon it is only observed when the constitutive isoform occurs and therefore cannot be tested for association with the skipping event using the EST data. Furthermore, there is only one EST that captures the exon skipping event, denoted by junction 334716 in ASAPII. There are also several cases of known splicing polymorphisms that could be detected from ESTs but not from the exon array data (Table 2). The gamma-aminobutyric acid (GABA) receptor, rho 1, gene (*GABRR1*), for example, was previously shown to have a SNP (rs4590242), located in the acceptor site that promotes use of an alternate NAGNAG acceptor [39]. We detected this srSNP in the genomic data and EST data provided evidence of its effect on splicing with an mSNP rs12200969, (p-value = 0.03). However, due to the lack of a probe that coincides exactly with the end of the exon, the exon arrays were unable to detect this subtle alternative splicing event.

The probability of linkage disequilibrium of an srSNP and mSNP decreases with the distance that separates them. This limitation is highlighted by the failure to associate several transcribed SNPs (rs3093906, rs3093905, rs3093921, rs3093925, rs3093926, and rs3093927), located >5000 bp away from a putative allele-specific splicing event in the Ribonuclease P RNA component H1 gene (*PARP-2*). The ASAPII database contains the two alternate donor sites at this junction that are 39 bp apart and are supported by a total of 28 expressed transcripts, and two *PARP-2 *protein isoforms differing by 13 amino acids have been deposited in the SWISSPROT database [40]. We detected an srSNP (rs2297616) located at position 4 of the corresponding splice donor site and the exon-array data provide strong evidence for an association between the splicing index of a probeset that overlaps the 39 bp region between the alternative donor sites and the genotype of this SNP (p-value = 2 × 10^-5^).

In previous work we used a heuristic method to find associations between SNPs mapped to ESTs and alternatively spliced isoforms in order to detect candidate allele-specific isoforms and to quantify the proportion of alternatively spliced genes that are spliced allele-specifically [10]. However, such associations can also occur because of normal regulation of alternative splicing. For example, consider an alternatively spliced gene for which ESTs occur in just two of the cDNA libraries in dbEST. Assuming that these libraries were constructed from the tissues of single individuals, it is possible that these individuals have different genotypes for an exonic SNP in the gene. If the alternative isoforms of the gene happen to be regulated in a tissue specific way and if the cDNA libraries are derived from different tissues then this could result in an association between the alleles of the SNP and the mRNA isoforms. This association can be highly significant if there are many ESTs of the gene in the two cDNA libraries in which it occurs. To circumvent this problem in our previous work, we took a maximum of two ESTs per cDNA library (one for each allele of the SNP from heterozygous libraries and just one from homozygous libraries). This caused a substantial loss of data and reduction in power to detect and quantify allele-specific mRNA splicing. In the present work we explicitly model the regulation of alternative splicing and make much better use of the available data.

Our results and previous reports [10,12] suggest that polymorphisms that affect splicing are common. This has important implications, not only for discovering the molecular bases of genetic diseases, but also for the study of alternative splicing. A gene cannot be confirmed to be alternatively spliced unless multiple isoforms are observed from the same allele. Until then the possibility remains that the alternative isoforms observed are polymorphic variants rather than alternatively spliced. Although we have found ample evidence for allelic differences in splicing, isoforms that result entirely from sequence variants might be less common. In the set of examples we report here, there is a relatively small proportion of cases in which the data suggest that the SI might be zero for some variants. Allele-specific splicing may be particularly important in the context of investigations of the regulation of alternative splicing [41,42]. Such investigations should ensure that multiple samples from the same tissue source are not treated as independent. This was an issue, in particular with early investigations of regulated splicing using EST sequences, in which multiple samples from the same cDNA library were used [42].

Regulation of splicing is incompletely characterized and additional *cis *elements that regulate splicing are still being discovered [43]. A limitation of the current study is that the srSNP candidates are restricted to a subset of well characterized *cis*-acting splice regulatory elements (donor and acceptor sites, polypyrimidine tract, branch points, and some exonic splicing elements). The phosphomannomutase 2 gene (*PMM2*), for example, which has allele-specific skipping of exon 5 due to a SNP that disrupts an ESE composed of (GAR)n repeats [44], where R is a purine, was detected using the EST data (p = 0.003); however, we could not identify an srSNP because the disrupted ESE is not detected by ESEfinder. Polymorphisms not found in *cis*-regulatory regions can also result in apparent allele-specific splicing if they introduce premature termination codons (PTC) [45] and cause differential nonsense-mediated decay of alternative alleles. Such SNPs are not included in our srSNP database. We have also restricted our analysis to single nucleotide polymorphisms but allele-specific splicing could be due in many cases to other types of polymorphisms such as insertions and deletions [46].

In the majority of the examples of allele-specific splicing we have detected, the difference in splicing is quantitative rather than qualitative. This can occur for a gene that is alternatively spliced, but for which a polymorphism exists that affects the proportions of alternative isoforms produced. In some cases, particularly for common polymorphisms, the size of the effect on SI can be relatively small, but still highly significant because of the relatively large number of individuals in each genotype group. In other cases, e.g., the alternative isoforms of the *OAS1 *gene shown in Figure 3, the SI associated with one genotype may be much greater than for the other genotypes. In general, the size of an effect on SI sufficient for an effect on phenotype is likely to vary substantially from transcript to transcript. Consistent with what has been observed previously for *cis*-acting polymorphisms with a quantitative effect on splicing [9], for the majority of the probesets for which SI was significantly associated with SNP genotype, the SI value of the heterozygote was intermediate to the SI of the two homozygotes. In 904 (69%) of 1,318 associations (corresponding to 1,083 different srSNPs) for which heterozygote and both homozygote cell-lines for the SNP were available, the SI of the heterozygote had an intermediate value. This was the case for all of the 148 stronger associations (that remained significant using a family-wise error rate of 0.05).

Loci that affect splicing might be termed splicing quantitative trait loci (sQTLs), by analogy with expression quantitative trait loci (eQTLs) that have become the subject of significant interest [47]. Although the heritability of individual SI values is, on average smaller than the heritability of overall transcript expression level, our results suggest that a greater proportion of genes show heritable splicing of at least one region of the transcript, than heritability of overall gene expression level. In this study we have attempted to identify only *cis-*acting sQTLs. *Trans-*acting sQTLs are also likely to exist, particularly at genes that are involved in regulating alternative splicing, but the ratio of *trans *to *cis *acting variants may be much smaller for sQTLs than for eQTLs, because of the relatively more complex regulation of transcription initiation compared to splicing. We have taken a candidate SNP approach to detecting splicing polymorphisms. With the availability of whole-genome exon array data it is also possible to adopt a less directed approach analogous to methods that have been used previously to detect expression quantitative trait loci [47]. Each probeset could be tested for association with every SNP that overlaps the transcripts to which it belongs. However, because of the multiplicity of probesets per gene this would result in a very large number of tests and would be likely to yield a much larger set of candidates, but potentially a set with lower specificity and for which interpretation is more difficult. Although we could identify a large number of candidate splicing polymorphisms by combining genomic, EST and exon array data, many of which were strongly associated with splicing, confirmation of causal relationships between human sequence variants will require experiment, probably involving mutagenesis so that relative isoform abundance can be compared between alternative alleles against an identical genetic background.

## Conclusion

We have carried out a genome-wide survey of human polymorphisms that are likely to affect mRNA splicing. We developed a maximum likelihood approach to test for evidence of allele-specific splicing from EST data. This was complemented by an analysis of exon array data in the public domain, generated from cell lines that were genotyped as part of the HapMap project. For each of the putative splicing polymorphisms identified in the genome-wide scan, we tested for an association between splicing index and SNP genotype and corroborated 1,185 examples at a false discovery rate cut-off of 0.1. We discuss advantages and disadvantages of alternative high-throughput methods to detect allele-specific splicing and report that a higher proportion of transcripts show evidence of heritability of mRNA splicing of at least some part of the transcript, than show evidence of gene expression heritability at the whole transcript level. Our results underscore the importance of mutations that affect splicing for understanding human phenotypes and genetic diseases and provide a resource that can be used to help assess the effects of human polymorphisms on mRNA splicing.

## Methods

### Identification of srSNPs

We downloaded known transcripts, chromosomal genomic data and SNP and exon tables from Ensembl version 36 [26], which is based on NCBI Genome build 35. Genes and SNPs that mapped to multiple locations on the genome were discarded. Introns were inferred from the exon genomic coordinates obtained from Ensembl. SNP positions relative to the Ensembl exons and introns were identified via genomic coordinates. SNP positions relative to exon/intron junctions were also determined for isoforms obtained from the ASAPII database.

Published tools for detecting splicing regulatory elements were either requested from authors or downloaded from their respective sites. We extracted 9 nucleotides from the donor splice sites and 23 nucleotides from the acceptor splice sites as required by the maximum entropy algorithm of Yeo and Burge, 2004 [18]. Scores for each pair of alternate alleles were then calculated [18]. We also identified an inflated frequency of SNPs at the G base of the canonical AG acceptor site which has been previously identified as a sequencing artifact [48]. We therefore restricted our analysis to validated SNPs using the information from dbSNP125 in the Ensembl database.

The ESEfinder tool [19] is designed to predict four ESEs: SC35, ASF2, SRp55, and SRp40. ESEfinder uses a position specific weight matrix. An ESE is considered to have a pre-defined length, *m*, and a recommended minimum score *S*. For each SNP we extracted *m*-1 nucleotides up- and downstream of the SNP. We then calculated the ESE scores for each of the contiguous length *m *subsequences of this sequence. The highest score for each SNP allele was retained if at least one of the scores was above *S *and the other below *S*. Although some strong ESEs can influence splicing at a distance of several kilobases [49], functional ESEs are most abundant in close proximity to splice junctions of internal exons [50]. We therefore restricted our analysis to ESEs located within 200 bps of exon-intron junctions of internal exons. Branch point scores for pairs of alternate SNP alleles were computed using Perl scripts provided by Kol et al., 2005 [24].

### Identification of mSNPs

We downloaded pre-computed EST and SNP genomic locations from the UCSC Genome Browser [51], which is based on NCBI genome assembly 36. ESTs and SNPs that mapped multiple times onto the genomic sequence and ESTs for which less than 90% of the sequence mapped to the genome were discarded. We used SNP and EST genomic coordinates to identify the SNP allele corresponding to each EST overlapping the SNP position. ASAPII [27], a database of alternatively spliced gene clusters, was downloaded on 9/11/2006. This data included gene and exon genomic locations based on NCBI genome assembly 35 as well as alternative mRNA isoforms (represented by conflicting exon junction pairs) mapped to ESTs.

### Models of regulated and allele-specific splicing

For a given allele, A, of an alternatively spliced gene with alternative splice isoforms S_1 _and S_2_, let *x *represent the proportion of isoform S_1 _produced from allele A in a cDNA library. We assume that *x *is constant for a given allele and library, but may vary across alleles and/or libraries. The purpose of the model is to determine, using data from several libraries (in which alternative transcript isoforms may be differentially regulated and have different relative expression levels), whether *x *shows significant variation across alleles.

Consider cDNA library *i *with *N *transcripts from allele A, of which we observe *a*_*i *_ESTs that map to S_1 _and *b*_*i *_= *N-a*_*i *_ESTs that map to S_2_. Because *a*_*i *_is binomially distributed with binomial parameter *x*, we use the beta distribution (conjugate to the binomial) to describe the probability density of *x*. We share this distribution across all libraries but not necessarily across the two alleles. Thus the values of *x *for separate libraries are modeled as independent draws from the distribution *f(x*, *α*_A_, *β*_A_*) *for allele A and *f(x*, *α*_B_, *β*_B_*) *for allele B, where *f(x*, *α*, *β) *is the beta function with parameters *α *and *β*.

The likelihood of the data from allele A observed in library *i *can now be expressed as

(1) L(D|iαA,βA)=∫01xai(1−x)bif(x,αA,βA)dx

The likelihood of the data observed in all cDNA libraries is a product over terms such as this, and the *α *and *β *parameters can be estimated by optimizing the likelihood for the combined data set.

An analytical solution to the integral of equation 1 exists, resulting in the following expression for the likelihood of the complete data for a pair of alternate isoforms and SNP alleles:

(2) $$L\left(D|\alpha_{A},\beta_{A},\alpha_{B},\beta_{B}\right)=\prod_{i}\frac{\Gamma \left(\alpha_{A}+\beta_{A}\right)\Gamma \left(a_{i}+\alpha_{A}\right)\Gamma \left(b_{i}+\beta_{A}\right)\Gamma \left(\alpha_{B}+\beta_{B}\right)\Gamma \left(c_{i}+\alpha_{B}\right)\Gamma \left(d_{i}+\beta_{B}\right)}{\Gamma \left(\alpha_{A}\right)\Gamma \left(\beta_{A}\right)\Gamma \left(a_{i}+b_{i}+\alpha_{A}+\beta_{A}\right)\Gamma \left(\alpha_{B}\right)\Gamma \left(\beta_{B}\right)\Gamma \left(c_{i}+d_{i}+\alpha_{B}+\beta_{B}\right)}$$

where *a*_*i*_, *b*_*i *_are the numbers of ESTs in cDNA library *i *that map to allele A and splice junctions S_1 _and S_2 _respectively and *c*_*i*_, *d*_*i *_are the corresponding EST counts for allele B. The maximum likelihood parameter estimates were obtained by optimizing the likelihood using Powell's method [52].

For the null model, we impose the restriction that *α*_A _= *α*_B _and *β*_A _= *β*_B_, such that both alleles are considered to be sampled from the same distribution (no allele-specific effect). To model allele-specific splicing (the alternative model), we allow *α*_A _≠ *α*_B _and estimate separate beta distributions for the alternate alleles of a SNP (we keep the constraint *β*_A _= *β*_B _because we found that this model already has sufficient freedom to model the desired effect and adding another degree of freedom was unnecessary). If the null model can be rejected in favour of the alternative model (using the likelihood ratio test) we conclude that there is evidence of allele-specific splicing.

### Simulations

We constructed 1000 random replicates of the EST data such that, for every SNP, the number of libraries derived from each genotype of the SNP was identical to the real data. Each library in the simulated data was assigned a genotype, with a probability proportional to the number of libraries of that genotype in the real data (this proportion was adjusted as each simulated library was assigned a genotype). For each library, the total numbers of ESTs derived from each isoform was constrained to be the same as in the real data. For heterozygous libraries ESTs were assigned to alternative SNP alleles with equal probability.

### Analysis of Affymetrix exon arrays

Whole genome exon data generated using the Affymetrix Human Exon 1.0 ST array by Huang et al. [25] were obtained from the Gene Expression Omnibus [29]. These data were generated from 166 cell lines for which genome-wide genotype data are available through the HapMap project [16]. All probes that overlapped with SNPs from dbSNP were removed because these may affect hybridization and cause artifactual association [12]. The exon array data was processed using the Affymetrix Power Tools [53]. For all probesets we estimated expression level in each cell-line using the Plier Sketch algorithm and estimated detection above background probabilities using DABG [53]. For the meta-probeset (transcript) level expression we used only the high confidence (or 'core') probesets from the array to avoid inaccuracy caused by the inclusion of computationally predicted probesets [12]. For each probeset that mapped to a meta-probeset, the splicing index (SI) was calculated by dividing the probeset expression estimate by the estimate of the transcript-level expression in each cell line. For non-core probesets that mapped to core as well as non-core meta-probesets the core meta-probeset expression estimate was used.

For each srSNP we used a robust linear model to test for an association between the SI of all probes within 1 kb of the probe and SNP genotype, using only unrelated individuals (i.e parents from the parent-child trios) and treating HapMap population as a covariate. We used Holm correction (with significance level 0.05) to control the family-wise error rate and establish a high-confidence or conservative set of probes with allele-specific SI. A false detection rate correction (also with cut-off set to 0.05) was also used to generate a larger set of events that includes a small proportion of false positive inferences. All statistical analyses were performed using the R statistical computing environment [31,32].

## Abbreviations

EST: Expressed Sequence Tag, SNP: Single Nucleotide Polymorphism, srSNP: splicing regulatory SNP, mSNP: marker SNP, BP: Branch point, ESE: Exonic splice enhancer, ANOVA: analysis of variance, PTC: Premature termination codon, sQTL: splicing quantitative trait locus, eQTL: expression quantitative trait locus, SI: splicing index

## Authors' contributions

CS conceived and supervised the project, analyzed the exon array data and designed the models of allele-specific splicing for EST data with input from KS. VN carried out the EST and SNP analysis and was also responsible for the integration of the different analyses with input from BL. KaS and ChS provided input into the direction of the research. All authors contributed towards writing and editing of the manuscript.

## Supplementary Material

Additional file 1 Results of genome-wide scan of polymorphisms in splicing-regulatory regions. **Exon-array diagrams**. Diagrams illustrating evidence of allele-specific splicing from exon-array data for 1,185 srSNPs are available from . Click here for file

Additional file 2 Allele-specific splicing candidates inferred from EST data. **Exon-array diagrams**. Diagrams illustrating evidence of allele-specific splicing from exon-array data for 1,185 srSNPs are available from . Click here for file

Additional file 3 Allele-specific splicing candidates inferred from exon array data. **Exon-array diagrams**. Diagrams illustrating evidence of allele-specific splicing from exon-array data for 1,185 srSNPs are available from . Click here for file

Additional file 4 Length differences and frame preservation of allele-specific isoform pairs. **Exon-array diagrams**. Diagrams illustrating evidence of allele-specific splicing from exon-array data for 1,185 srSNPs are available from . Click here for file
