# Supplementary material for: Genome-wide survey of allele-specific splicing in humans
Source: BMC Genomics. 2008 Jun 2;9:265. doi: 10.1186/1471-2164-9-265 (PMC2427040; doi:10.1186/1471-2164-9-265)
Supplement: Additional file 1 — Results of genome-wide scan of polymorphisms in splicing-regulatory regions. Exon-array diagrams. Diagrams illustrating evidence of allele-specific splicing from exon-array data for 1,185 srSNPs are available from . [file 1471-2164-9-265-S1.doc]

**Supplementary Notes**

**Length difference between allele-specific splicing isoforms**

We calculated the difference in length between putative allele-specific splicing isoforms that we identified using the EST data and compared this to isoform length differences inferred from the full set of mutually exclusive alternatively spliced isoforms pairs from ASAPII. We distinguished between the allele-specific isoforms inferred with high-confidence (with a false discovery rate after taking account of multiple hypothesis tests less than 0.2; see main manuscript) and low-confidence allele-specific isoforms (with an uncorrected p-value less than 0.05). We observed the smallest length difference among the allele-specific isoforms inferred with high confidence (Table S1).

**Table S1: Mean (and standard error) of the differences in alternative isoform lengths (p-values shown are for comparisons with all isoform pairs using a t-test).**

|  | All isoform pairs **mean (standard error)** | Isoform pairs with weak evidence of allele-specific splicing | Isoform pairs with strong evidence of allele-specific splicing |
| --- | --- | --- | --- |
| **Cassette** | **139.16** (1.13) | **130.57** (3.21)  p = 0.012 | **116** (11.36)  p = 0.052 |
| **5’** | **108.58** (2.98) | **87.60** (9.01)  p = 0.029 | **72.33** (20.67)  p = 0.17 |
| **3’** | **147.42** (5.54) | **167.45** (37.92)  p = 0.60 | **53.89** (23.44)  p = 0.0053 |
| **All** | **134.66** (1.13) | **128.23** (3.95)  p = 0.12 | **100.21** (9.99)  p = 0.0013 |

**Frame preservation**

For each pair of mutually exclusive alternatively spliced isoforms from ASAPII located within predicted coding regions we determined whether the difference in length between alternative isoforms was a multiple of 3 nucleotides (and therefore frame preserving). The rate of frame preservation that we observe was similar to a previous estimate of 40% reported by Modrek et al [1]. The proportions of alternative splicing events that are frame preserving was slightly higher for events with strong evidence of allele-specific splicing (Table S2) but the difference was not statistically significant.

**Table S2: Proportions of frame-preserving alternative splicing events**

|  | All isoform pairs | Isoform pairs with weak evidence of allele-specific splicing | Isoform pairs with strong evidence of allele-specific splicing |
| --- | --- | --- | --- |
| **Cassette** | **41.42%**  5311/12749 | **41.44%**  230/555 | **47.37%**  9/19 |
| **5’** | **44.27%**  1286/2905 | **38.94%**  37/95 | **100%**  2/2 |
| **3’** | **42.87%**  722/1684 | **42.59%**  23/54 | **60%**  3/5 |
| **All** | **42.09%**  6696/15908 | **41.19%**  290/704 | **53.87%**  14/26 |

Reference List

1. Modrek B, Resch A, Grasso C, Lee C: **Genome-wide detection of alternative splicing in expressed sequences of human genes.** *Nucleic Acids Res* 2001, **29:** 2850-2859.
